# High‐Temperature Superconductivity of Thermodynamically Stable Fluorite‐Type Hydrides at Ambient Pressure

**DOI:** 10.1002/advs.202512696

**Published:** 2025-09-04

**Authors:** Hongyu Huang, Xiaoyan Yan, Min Wang, Mingyang Du, Hao Song, Zhao Liu, Defang Duan, Tian Cui, Xinmiao Wei

**Affiliations:** ^1^ Institute of High Pressure Physics School of Physical Science and Technology Ningbo University Ningbo 315211 P. R. China; ^2^ College of Physics Jilin University Changchun 130012 P. R. China

**Keywords:** first principles calculation, high pressure, hydrides, superconductivity

## Abstract

Hydride superconductors have attracted significant attention, yet achieving superconductivity at ambient pressure remains a key challenge. Here, a family of high‐*T*_c_ (superconducting critical temperature, *T*_c_) hydrides based on the fluorite‐type AXH_8_ structure, exhibiting thermodynamic and dynamic stability at low to atmospheric pressure, is proposed. Through comprehensive screening of 150 ternary systems, eight stable hydrides below 35 GPa are identified. Notably, AcRhH_8_ and BaRhH_8_ show ambient‐pressure stability, while AcIrH_8_ and BaIrH_8_ are stable at 3 GPa, demonstrating great potential for practical applications. Several compounds surpass the liquid‐nitrogen *T*_c_ threshold, including AcRhH_8_ (78 K at 0 GPa), LaRhH_8_ (94 K at 24 GPa), LaOsH_8_ (83 K at 35 GPa), and CeOsH_8_ (106 K at 31 GPa). Mechanistic analysis reveals that large‐radius A‐site atoms serve as electron donors, stabilizing the framework and promoting weak covalent connections among [XH_8_]^n−^ anionic units. This leads to a 3D weak covalent network that enhances electron delocalization, increases the density of states at the Fermi level, and strengthens electron–phonon (EPC). Moreover, a linear correlation between minimum ELF at H_8_ tetrahedral connections and EPC constant further highlights the role of hydrogen‐framework charge delocalization in boosting superconductivity.

## Graphical abstract

Fluorite‐type AXH_8_ hydrides comprise H_8_ units formed by small‐radius X elements and hydrogen, linked into a 3D weak covalent network. Large‐radius A atoms act as electron donors, stabilizing the lattice and enhancing delocalization. A linear correlation between minimum ELF at H_8_ connections and electron–phonon coupling λ underpins their superconductivity, exemplified by ambient‐pressure AcRhH_8_ (78 K).

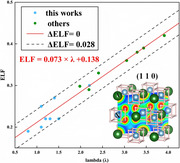

## 1 Introduction

Since the discovery of superconductivity in mercury by Heike Kamerlingh Onnes in 1911,^[^ ^1^ ^]^ the pursuit of high temperature superconductivity has remained a central theme in condensed matter physics. The BCS theory links the superconducting transition temperature (*T*_c_) to lattice vibrations via electron–phonon interactions.^[^ ^2^ ^]^ Since phonon frequencies, and hence the Debye temperature, scale inversely with the square root of atomic mass, *T*_c_ exhibits a similar isotope effect in conventional superconductors. This insight positioned hydrides as promising candidates for achieving high *T*_c_. This theoretical insight sparked a wave of research into hydrogen‐rich compounds. Ashcroft proposed that metallic hydrogen could exhibit room‐temperature superconductivity^[^ ^3^ ^]^; however, the extreme pressures required for hydrogen metallization posed substantial experimental challenges. To overcome this, in 2004, he introduced the concept of chemical precompression, suggesting that alloying hydrogen with other elements could lower the metallization pressure and make high *T*_c_ superconductivity more accessible.^[^ ^4^ ^]^

The breakthrough in 2014, when superconductivity in H_3_S was predicted theoretically and soon confirmed experimentally at 203 K near 250 GPa,^[^ ^5^ ^]^ marked a milestone in the field of high‐pressure superconductors. Subsequently, binary hydrides represented by LaH_10_ were successfully predicted and experimentally synthesized.^[^ ^6^, ^7^, ^8^, ^9^, ^10^ ^]^ Recently, experimentalists successfully measured the superconducting gap of H_3_S using tunneling spectroscopy.^[^ ^11^ ^]^ The results confirmed s‐wave pairing and a gap behavior consistent with BCS theory, offering strong evidence for a phonon‐mediated mechanism and validating the accuracy of the original theoretical models. These advances highlight both the deepening understanding of hydride superconductivity mechanisms and the proven accuracy of theory predictions. Nevertheless, the requirement for extreme pressures remains a major obstacle to practical implementation. To address this, research has increasingly turned toward ternary hydrides.^[^ ^12^, ^13^, ^14^, ^15^, ^16^, ^17^, ^18^, ^19^ ^]^ Although several ternary hydrides, such as (La,Ce)H_9, 10_,^[^ ^20^ ^]^ and (La,Y)H_10_,^[^ ^21^ ^]^ have demonstrated near room temperature, they still require significant compression. While ternary hydrides have expanded the chemical space for superconductivity, achieving ambient or near‐ambient pressure superconductivity remains a formidable challenge.

Driven by the pursuit of high‐temperature superconductors at near‐ambient pressures, attention has turned to hydrides capable of maintaining high *T*_c_ under milder conditions. A key breakthrough was the synthesis of LaBeH_8_, with a *T*_c_ of 110 K at 80 GPa,^[^ ^22^, ^23^ ^]^ marking progress toward lower‐pressure superconductivity. This spurred predictions of related compounds such as ThBeH_8_,^[^ ^24^ ^]^ AcBeH_8_,^[^ ^25^ ^]^ LuBeH_8_,^[^ ^26^ ^]^ YbBeH_8_,^[^ ^26^ ^]^ and quaternary systems like CaTh_3_Be_4_H_32_^[^ ^27^ ^]^ and LaThBe_2_H_16_.^[^ ^28^ ^]^

Several low or ambient pressure hydrides have also been reported, including RbTlH_3_ (170 K at 4 GPa),^[^ ^29^ ^]^ CsBH_5_ (83 K at 1 GPa),^[^ ^30^ ^]^ ThBeH_8_ (113 K at 7 GPa),^[^ ^24^ ^]^ KB_2_H_8_ (146 K at 12 GPa),^[^ ^31^ ^]^ LuN_4_H_11_ (100 K at 20 GPa),^[^ ^32^ ^]^ BaBiH_8_ (94 K at 20 GPa),^[^ ^33^ ^]^ and MgN_2_H_8_ (105 K at 30 GPa).^[^ ^34^ ^]^ Some, like PdCuH_2_,^[^ ^35^ ^]^ and MgHCu_3_,^[^ ^36^ ^]^ exhibit superconductivity at ambient pressure, while others such as Mg_2_RhH_6_,^[^ ^37^, ^38^, ^39^ ^]^ Mg_2_IrH_6_,^[^ ^37^, ^38^, ^39^ ^]^ and Li_2_AuH_6_^[^ ^40^ ^]^ show promising *T*_c_ over 50 K. At moderate pressures, LiHeH_6_ (177 K at 90 GPa)^[^ ^41^ ^]^ NaKH_12_ (234 K at 60 GPa)^[^ ^42^ ^]^ and LaTh_3_H_24_ (198 K at 50 GPa)^[^ ^43^ ^]^ were predicted successfully.

In this study, we carried out high‐throughput calculations on 150 ternary hydride systems. Our results reveal that compounds with the formula AXH_8_ (A = La, Ba, Ac, Ce; X = Rh, Ir, Os) exhibit robust dynamical and thermodynamic stability, with four structures remaining stable under ambient conditions. Remarkably, AcRhH_8_ not only maintains ambient pressure stability but also achieves a *T*_c_ surpassing the liquid nitrogen temperature threshold (*T*_c_ > 77 K). Detailed electronic structure analysis shows that large radius elements act as electron donors stabilizing the lattice, while the small radius transition metal and hydrogen atoms construct the [XH_8_]^n−^ anionic units, which form a 3D weakly covalent network. This network, characterized by flat band features and van Hove singularities at the Fermi level, enhances electron–phonon coupling. Furthermore, the minimum electron localization function (ELF) at the tetrahedral connections of the H_8_ units exhibits a strong linear correlation with the electron–phonon coupling (EPC) constant λ, expressed as ELF = 0.073λ + 0.138. This highlights a direct relationship between electron phonon interactions and the delocalized charge distribution within the hydrogen alloy framework. The findings deepen our understanding of the microscopic mechanisms governing superconductivity in these hydrides and offer a theoretical basis for the rational design of low pressure, high temperature superconductors. Furthermore, the work provides practical insights that may guide future experimental efforts in the search for novel hydrogen‐based superconducting materials.

## 2 Results and Discussion

**Figure 1:**
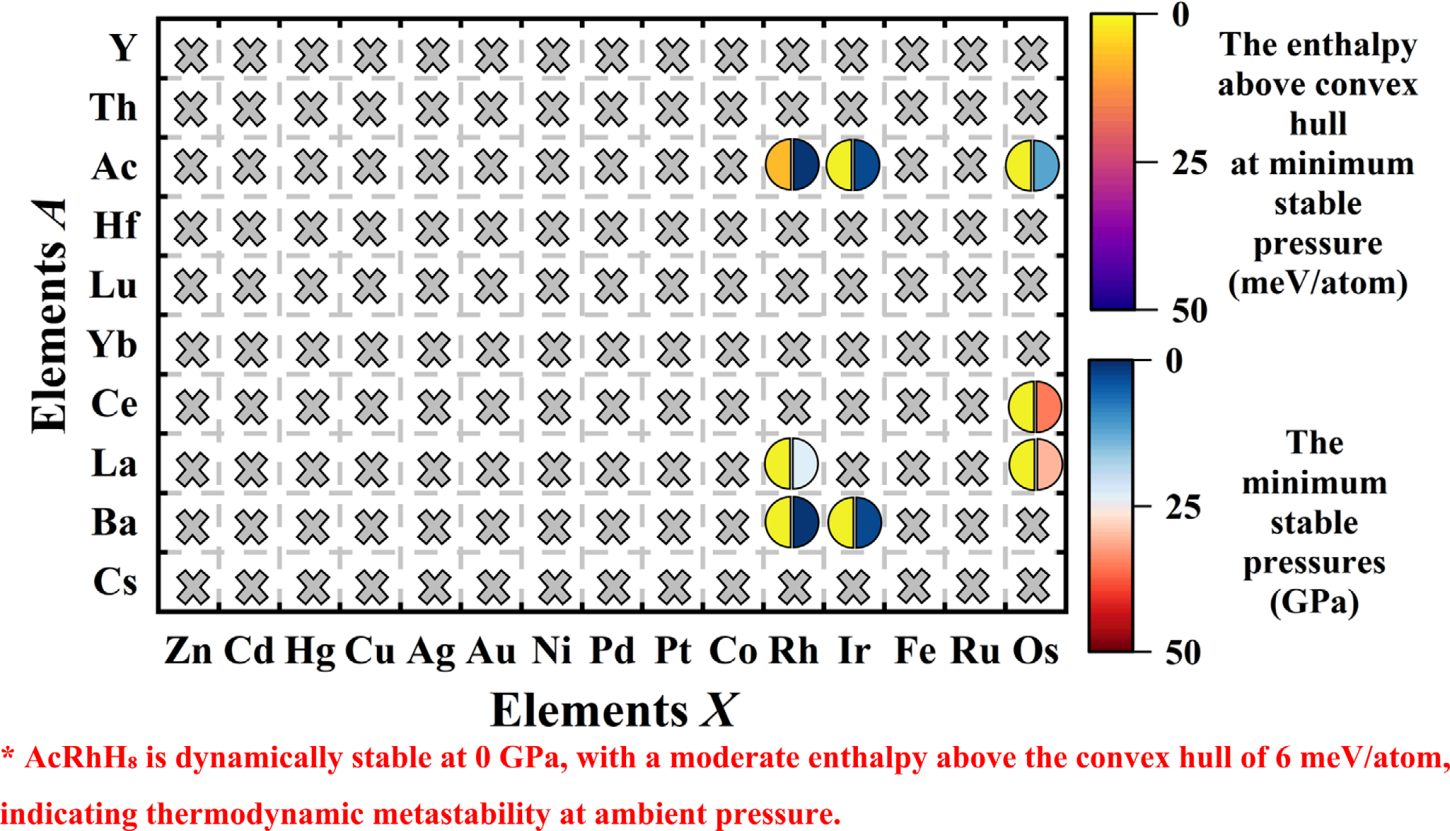
Stability distribution of AXH₈ hydrides formed by combining large‐radius elements (A) with small‐radius transition metals (X). Crossed symbols represent dynamically unstable phases, while semicircles denote dynamically stable compounds. The right half of each semicircle indicates the lowest pressure at which the structure is dynamically stable, and the left half shows the enthalpy above the convex hull at that pressure. Notably, AcRhH₈ exhibits dynamical stability at 0 GPa with an enthalpy of 6 meV/atom, suggesting thermodynamic metastability at ambient pressure, despite becoming fully stable at 10 GPa.

In this study, inspired by the fluorite type LaBeH_8_ structure, we systematically designed a series of AXH_8_ compounds through elemental substitution. As shown in the **Figure** **1**, the A elements were chosen from large‐radius elements (including Ac, La, Th, Hf, Ba, Ce, Cs, Lu, and Yb), and the X elements were selected from small radius elements (covering groups IB‐IIB and VIIB‐XB). Through high‐throughput calculations, we thoroughly investigated the structural stability, thermodynamic properties, and superconducting performance of these compounds within the pressure range of 0–50 GPa. As shown in Figure 1, we calculated a total of 150 structures and successfully screened out 8 dynamically stable fluorite type hydrides: AcRhH_8_, AcIrH_8_, AcOsH_8_, BaRhH_8_, BaIrH_8_, LaRhH_8_, LaOsH_8_, and CeOsH_8_.

In AXH_8_ compounds, the A element occupies the 4a Wyckoff site with fractional coordinates (0,0,0), the X element is located at the 4b Wyckoff site with fractional coordinates (0.5,0,0), and the H atoms are distributed at the 32f Wyckoff site with fractional coordinates (0.34372,0.84372,0.15628). In this structure, H atoms are primarily coordinated with the X element in cubic or tetrahedral arrangements, forming anionic‐like groups [XH_8_]^n−^ and constructing a hydrogen alloy framework with high symmetry.

**Figure 2:**
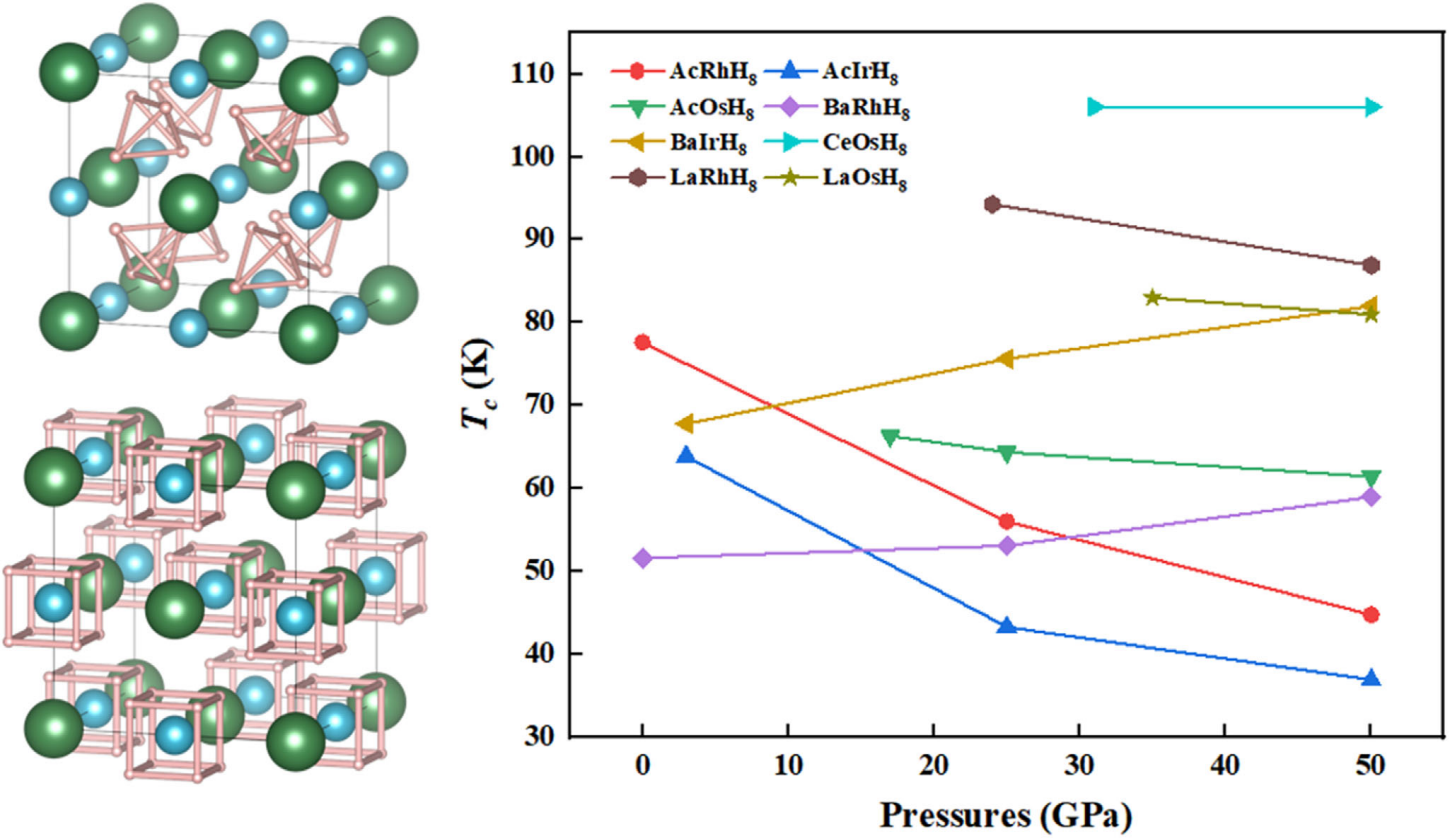
The left panel displays the crystal structure of AXH_8_ hydrides, highlighting the presence of two distinct hydrogen units. The upper left structure shows H atoms forming tetrahedral units, while the lower‐left structure reveals cubic H units, both of which play a vital role in the material's superconducting properties. The large green spheres represent A site atoms, the medium blue spheres denote X site atoms, and the small pink spheres correspond to H atoms. The right panel presents the superconducting transition temperatures of eight AXH_8_ hydrides as a function of pressure (0‐50 GPa).

We evaluated the thermodynamic stability of the identified structures using the AIRSS method.^[^ ^44^ ^]^ At their lowest stable pressures, seven of the eight compounds were located on the convex hull (Figure S1, Supporting Information), indicative of good thermodynamic stability. AcRhH_8_ was slightly above the convex hull at 0 GPa (6 meV/atom) and became fully thermodynamically stable at 10 GPa (Figure S1a, Supporting Information, suggesting favorable experimental synthesis prospects for all investigated structures. All candidate compounds demonstrated excellent dynamic stability, with AcRhH_8_, AcIrH_8_, BaRhH_8_, and BaIrH_8_ exhibiting dynamic stability near ambient pressure. As shown in **Figure** **2**, *T*_c_ exceeding liquid nitrogen temperature were observed for CeOsH_8_, LaRhH_8_, LaOsH_8_, and AcRhH_8_ at their lowest stable pressures. Pressure‐dependent *T*_c_ calculations (0, 25, 50 GPa) revealed that Tcs for BaIrH_8_ and BaRhH_8_ increased with pressure up to 50 GPa, while CeOsH_8_ showed minimal change, and other structures exhibited a moderate decrease.

**Figure 3:**
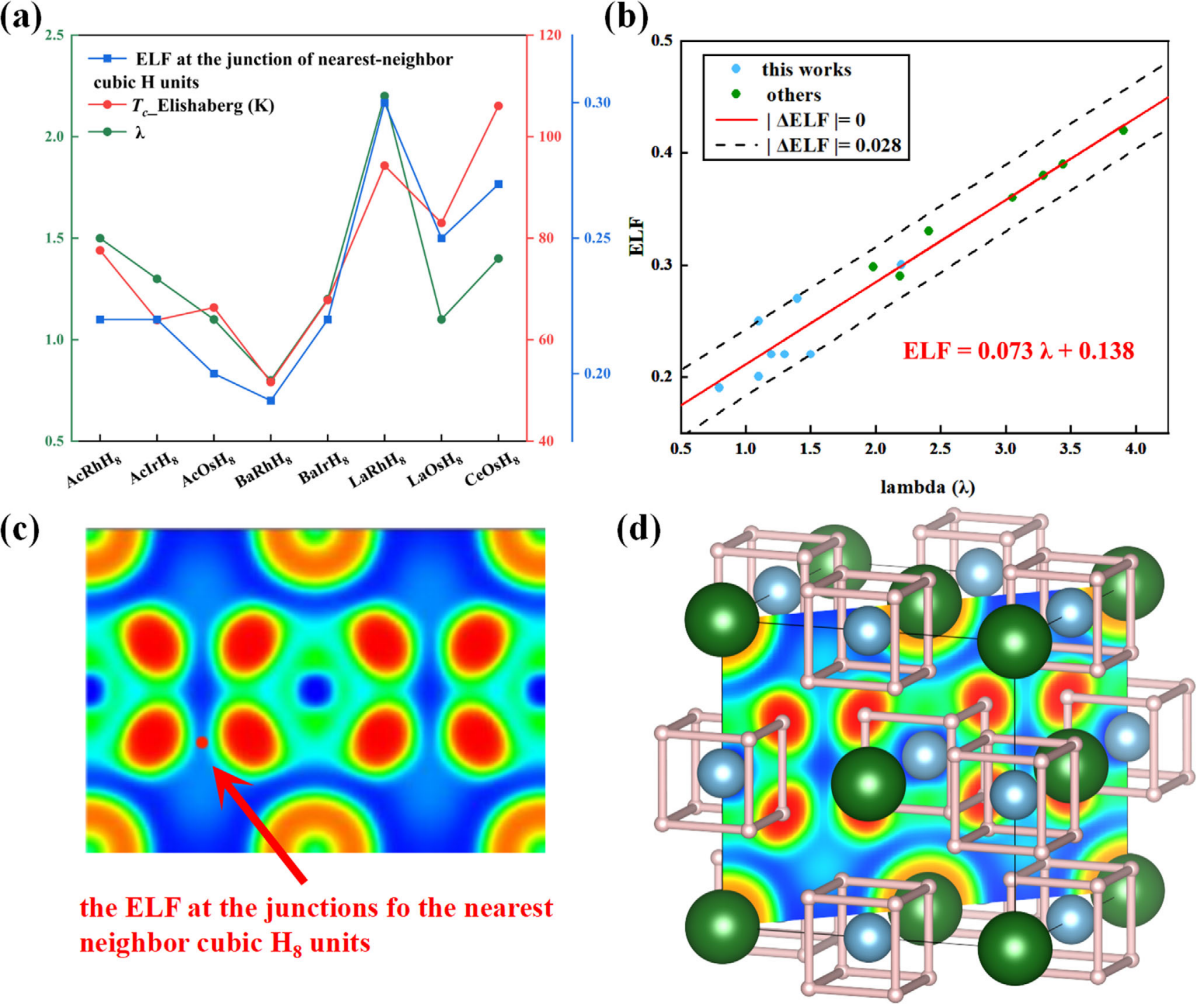
a) The relationship between the ELF at the junctions of the cubic units, the EPC parameter (λ), and the superconducting transition temperature calculated via Eliashberg theory; b) the linear correlation between the minimum ELF and λ. Light blue dots represent data from this work, while green dots denote results from previous studies. The red solid line corresponds to the fitted relationship ELF = 0.073 λ + 0.138, with dashed black lines indicating a deviation range of ± 0.028. c) ELF showing the cross section at the junctions between neighboring cubic H_8_ units. The red arrow indicates enhanced electron localization, suggesting possible inter‐unit interactions. d) Crystal structure composed of cubic H_8_ units (outlined in pink), with green and blue spheres representing A and X elements. The arrangement highlights the spatial distribution and connectivity of the H_8_ clusters within the lattice.

To understand the electronic properties of AXH_8_ structures, we performed a detailed analysis of ELF (**Figure** **3**b,c), the Bader charge distribution (Table S1, Supporting Information), and crystal orbital Hamilton population (COHP)^[^ ^45^ ^]^ (Figure S2, Supporting Information) at the lowest stable pressures. Bader charge analysis revealed that the large radius A elements act primarily as electron donors, contributing approximately 1.75 e^−^ to maintain system stability. The ELF analysis indicated significant electron localization around the A elements, strategically positioned at vertices and face centers, critical for lattice framework stability and mitigating high‐pressure distortion.

Further examination of the ELF distribution along the (1 1 0) plane provided deeper insight into internal electronic features. In these hydrides, the X elements and hydrogen atoms form anionic‐like [XH_8_]^n−^ groups with characteristic high electron localization around hydrogen, suggesting intra‐group covalent interactions. Critically, between adjacent anionic groups, the ELF distribution exhibits regions of lower electron localization, indicating a weak covalent bond network as illustrated in the Figure 3c. This network connects the otherwise isolated anionic units, forming a continuous electronic network.

To explore the universality of the relationship between the ELF at the tetrahedral connection of the H_8_ units and the λ in AXH_8_ structures, we analyzed eight structures from this work along with data from other studies on AXH_8_ systems. The results reveal a linear correlation between λ and the ELF at the H_8_ tetrahedral connection sites, described by the equation: ELF = 0.073λ + 0.138. This relationship indicates that the EPC strength in AXH_8_ structures is proportional to the minimum ELF value on the hydrogen alloy framework, highlighting a strong connection between electron–phonon interaction and the delocalized charge distribution.

The low ELF indicated a weak covalent network between adjacent anionic groups forms effective charge transport channels, thereby enhancing overall electron mobility. This bridging effect not only connects otherwise isolated anionic groups but also constructs a more extensive, anion‐dominated electron transport network. As a result, additional electron pathways emerge near the Fermi level, improving charge transfer efficiency. Moreover, the increased effective carrier density from these bridging electrons strengthens the EPC parameter λ, in agreement with the enhanced ELF signals observed within the anionic groups.

The COHP analysis further revealed that the small radius X elements occupy hydrogen antibonding orbitals, and weak X‐H covalent bonds are prominent near the Fermi level. This weak covalent component at the Fermi surface is a key factor in electron–phonon interaction and superconducting properties. The dominance of X‐H covalent bonds near the Fermi surface leads to stronger interactions during EPC, further boosting λ. This combined mechanism, where the A element facilitates the interconnected weak covalent network and the X‐H bonds near the Fermi level drive EPC, underlies the superconducting performance and structural stability of the AXH_8_ system.

**Figure 4:**
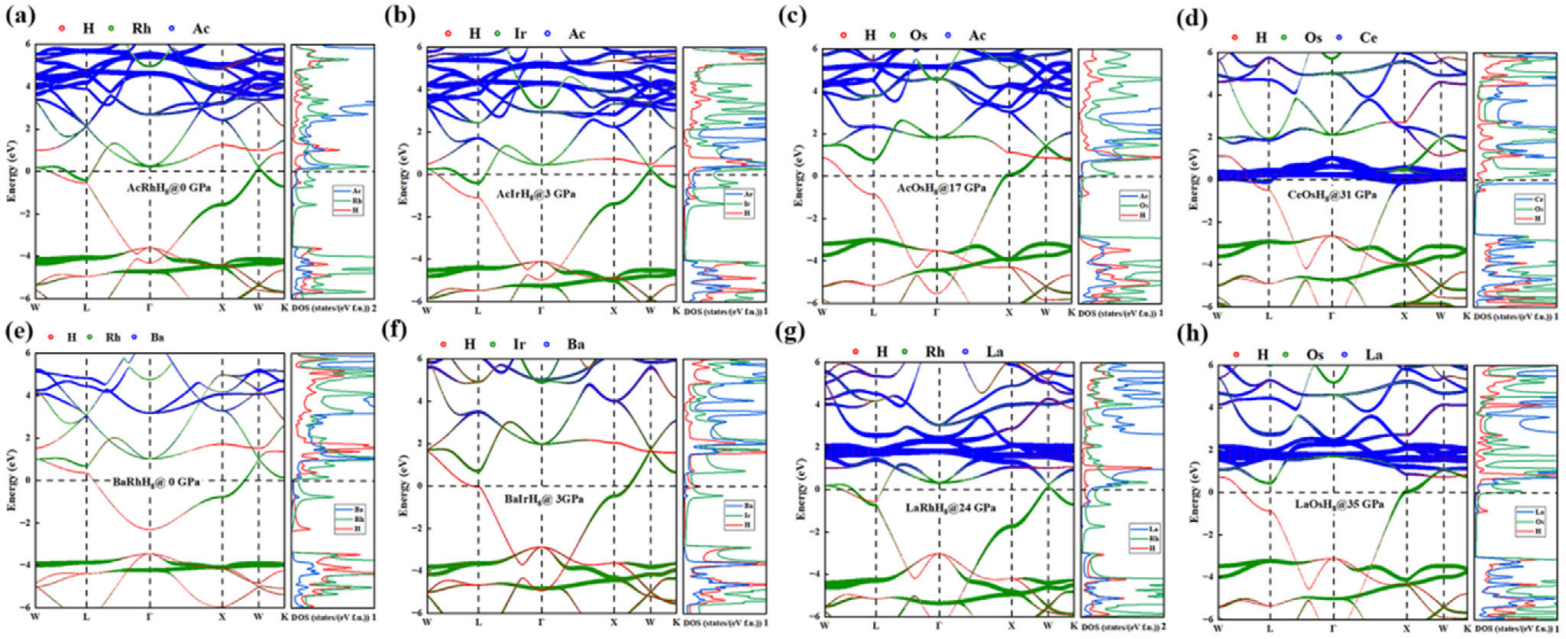
The electronic fat band structure and the projected density of states of a) AcRhH_8_, b) AcIrH_8_, c) AcOsH_8_, d) CeOsH_8_, e) BaRhH_8_, f) BaRhH_8_, g) LaRhH_8_, and h) LaOsH_8_ at their respective lowest dynamical stable pressures.

To understand the superconductivity mechanisms in AXH_8_ systems, we analyzed their electronic band structures and PDOS at stable pressures, as shown in **Figure** **4**. All structures exhibit clear metallic behavior, evidenced by bands crossing the Fermi level, which plays a crucial role in their superconducting properties.

A closer examination of the electronic states in the vicinity of the Fermi level highlighted a significant finding: the region near the Fermi level is primarily dominated by the contributions from the X elements (specifically Rh, Ir and Os) and the hydrogen atoms. In contrast, the A elements contribute less significantly to the electronic states in this crucial energy range, and consequently, their direct influence on the EPC is found to be relatively limited.

However, a pivotal observation for understanding the superconducting properties was the presence of flat bands and pronounced van Hove singularity (vHs) originating from the electronic states of the X and H elements directly at the Fermi surface. These electronic features are identified as key factors that significantly enhance the EPC. Specifically, the flat bands near the Fermi level correspond to regions where the electron velocity is low. This reduced velocity leads to an increased residence time of electrons in these regions, effectively increasing the local electron concentration at the Fermi surface, which in turn boosts the strength of the EPC. Furthermore, the vHs characterized by a localization and sharp increase in the density of states at specific energy points lead to a significant enhancement of the density of states near the Fermi level. This enhanced density of states facilitates a higher electron filling at the Fermi level, which is known to contribute to a larger EPC constant, denoted as λ.

Beyond the intrinsic electronic features, we also explored the impact of doping effects on the electronic structure and electron–phonon interaction. The effect of elemental doping offers a powerful means to optimize the electron–phonon interaction by precisely tuning the position of the Fermi level and the overall density of states. Moreover, the application of pressure plays a critical role by enhancing the orbital coupling between the hydrogen and X elements (Figure S4, Supporting Information). Pressure effectively tunes the electronic interactions between X and H elements by modifying their orbital hybridization near the Fermi level, further driving the EPC and consequently improving the superconducting performance.

This intricate interplay highlights a crucial point: the A element, through its role in donating electrons and facilitating the formation of the connected weak covalent network between anionic groups, works in synergistic effect with the electronic contributions of the X and H elements near the Fermi level. This collective synergy is found to be fundamentally critical for enhancing both the EPC and the superconducting transition temperature. Our comparative analysis of different ARhH_8_ systems provides compelling evidence for the effectiveness of elemental doping (Figure S4, Supporting Information). For instance, replacing the Ba element with Ac or La in the ARhH_8_ structure leads to an increase in the total number of electrons in the system. This increase results in an upward shift of the Fermi level, bringing it closer to the flat bands. This strategic shift significantly increases the electronic density of states near the Fermi surface, which in turn enhances the EPC constant λ, thereby promoting improved superconducting performance.

Indeed, in the structures exhibiting superior superconducting properties, specifically AcRhH_8_, LaRhH_8_, LaOsH_8_, and CeOsH_8_, the electronic band structure reveals that the flat bands and van Hove singularities are positioned even closer to the Fermi level. This enhanced proximity leads to a significantly higher electron concentration at the Fermi surface in these materials, which further amplifies the EPC strength. This amplified EPC is a direct consequence of the electronic structure and is a key factor in explaining why these particular structures exhibit superconducting transition temperatures that exceed that of liquid nitrogen.

**Figure 5:**
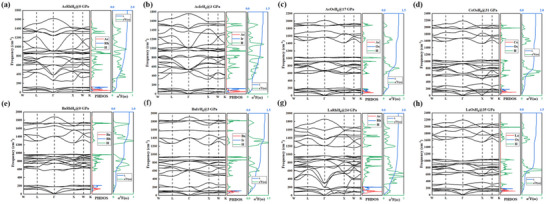
The calculated phonon band structure, PHDOS, EPC parameter λ, and Eliashberg spectral function α^2^F(ω) of a) AcRhH_8_, b) AcIrH_8_, c) AcOsH_8_, d) CeOsH_8_, e) BaRhH_8_, f) BaIrH_8_, g) LaRhH_8_, and (h) LaOsH_8_ at their lowest dynamical stable pressures.

Finally, we analyzed the phonon spectra of the AXH_8_ system at its lowest stable pressure to ascertain dynamic stability and vibrational characteristics. As illustrated in the **Figure** **5**, the absence of negative frequency modes confirmed dynamic stability under pressure. The phonon spectrum exhibits a clear separation into three distinct frequency regions. The low‐frequency modes are predominantly attributed to the vibrations of the heavy, large‐mass metal elements. These modes are reflective of the overall lattice rigidity and the fundamental stability of the crystal framework, acting as the backbone of the structure. Moving to the medium and high frequency regimes, the vibrational landscape is overwhelmingly dominated by the motion of the light hydrogen atoms. These high energy vibrations, intimately connected to the local environment and dynamics of hydrogen, are recognized as having a profound impact on the superconducting transition temperature.

Overall, a comprehensive examination of the phonon spectra demonstrates that the application of external pressure leads to a more compact lattice structure and a general upward shift in vibrational energies, as shown in Figure S5 (Supporting Information). The low‐frequency vibrations underscore the vital supportive role played by the massive elements in maintaining lattice stability. In contrast, the medium and high‐frequency vibrations, primarily driven by hydrogen dynamics and intrinsically linked to the weak covalent network bridging the anionic groups, exert a decisive influence on the EPC strength. These detailed findings offer a compelling theoretical foundation for both comprehending the superconducting mechanisms at play and for guiding future strategies aimed at optimizing the superconducting properties of these promising materials.

## 3 Conclusion

This study, based on first‐principles calculations, predicts and designs a series of fluorite‐type superconducting hydrides, including AcRhH_8_ (78 K at 0 GPa), LaRhH_8_ (94 K at 24 GPa), LaOsH_8_ (83 K at 35 GPa), CeOsH_8_ (106 K at 31 GPa), AcIrH_8_ (64 K at 3 GPa), BaRhH_8_ (52 K at 0 GPa), and BaIrH_8_ (68 K at 3 GPa). The results indicate that the synergistic coordination between large radius A and small radius X elements effectively stabilizes the AXH_8_ crystal structures, ensuring both thermodynamic and dynamic stability, and making them viable for experimental synthesis. Electronic structure analysis reveals that the minimum ELF at the tetrahedral linkages of the H_8_ anionic framework serves as a key node in the weak covalent network. There exists a strong linear dependence between the minimum ELF at the tetrahedral junctions of the H_8_ framework and the EPC strength λ, ELF = 0.073 λ + 0.138. This suggests that the ELF value at the tetrahedral linkages of the H_8_ anionic framework not only reflects the degree of electron localization but also serves as an effective predictor of EPC strength in AXH_8_ systems. The weak covalent bonding among X‐H anionic groups, combined with the electron bridging effect provided by A site elements, forms efficient charge transport pathways. The high‐frequency phonon modes dominated by light hydrogen atoms, together with the van Hove singularity near the Fermi level, synergistically enhance the superconducting transition temperature. Moreover, pressure tuning and A‐site elemental substitution can further optimize the electronic structure and EPC strength, thereby improving superconducting performance. This work not only provides a clear strategy for designing novel hydride superconductors at lower pressures but also proposes a microscopic, ELF‐based approach to effectively regulate EPC, laying a solid theoretical foundation for future experimental realization and discovery of hydrogen‐based high‐temperature superconductors.

## 4 Experimental Section

Crystal structure predictions for AXH_8_ compounds were conducted using the Ab Initio Random Structure Searching (AIRSS) method,^[^ ^44^, ^46^ ^]^ which generates random candidate lattices and relaxes them in parallel to efficiently identify low‐energy configurations. Initial relaxations were performed within the framework of density functional theory as implemented in CASTEP,^[^ ^47^ ^]^ employing the Perdew–Burke–Ernzerhof (PBE) functional under the generalized gradient approximation (GGA).^[^ ^48^ ^]^ A plane‐wave energy cutoff of 450 eV and a k‐point spacing of 0.07 × 2π Å^−1^ were adopted during the structure search to ensure computational efficiency. To accurately determine phase stabilities, promising structures were re‐optimized at higher precision within AIRSS, using an increased plane‐wave cutoff of 800 eV and a refined k‐point spacing of 0.03 × 2π Å^−1^. For consistency, relevant structures from the Materials Project database were also re‐relaxed under the same computational settings.

Electronic properties, including the ELF^[^ ^49^ ^]^ and Bader charge distributions,^[^ ^50^, ^51^ ^]^ were calculated using the Vienna Ab initio Simulation Package (VASP),^[^ ^52^ ^]^ employing the projector augmented‐wave (PAW) method with GGA‐PBE functionals. An 800 eV cutoff and dense Monkhorst–Pack meshes^[^ ^53^ ^]^ corresponding to a 0.03 × 2π Å^−1^ spacing were used, achieving enthalpy convergence within 1 meV per atom.

Phonon spectra and EPC properties were investigated using Quantum ESPRESSO.^[^ ^54^ ^]^ Self‐consistent field (SCF), phonon and Eliashberg spectral function α^2^F(ω) calculations were performed on 12 × 12 × 12 k‐point mesh and 4 × 4 × 4 q‐point meshes, with a plane‐wave cutoff of 90 Ry for wavefunctions to ensure reliable convergence of the EPC parameters. For structures identified as thermodynamically stable, EPC calculations were further refined using denser grids (12 × 12 × 12 for k‐points and 6 × 6 × 6 for q‐points). The superconducting transition temperatures were then estimated by solving the Allen‐Dynes modified McMillan equation^[^ ^55^ ^]^ and the self‐consistent solution of Eliashberg equations (scE)^[^ ^56^ ^]^ with the Coulomb pseudopotential of μ^*^ = 0.1 and 0.15.

## Conflict of Interest

The authors declare no conflict of interest.

## Supporting information



Supporting Information

## Data Availability

The data that support the findings of this study are available in the supplementary material of this article.
