# Supplementary material for: High‐Temperature Superconductivity of Thermodynamically Stable Fluorite‐Type Hydrides at Ambient Pressure
Source: Adv Sci (Weinh). 2025 Sep 4;12(44):e12696. doi: 10.1002/advs.202512696 (PMC12667446; doi:10.1002/advs.202512696)
Supplement: Supplementary file 1 — Supporting Information [file ADVS-12-e12696-s001.docx]

High-Temperature Superconductivity of Thermodynamically Stable Fluorite type Hydrides at Ambient Pressure

Hongyu Huang, Xiaoyan Yan, Min Wang, Mingyang Du*, Hao Song, Zhao Liu*, Defang Duan, Tian Cui*, Xinmiao Wei*

Hongyu Huang, Xiaoyan Yan, Min Wang, Mingyang Du, Hao Song, Zhao Liu, Tian Cui, Xinmiao Wei

Institute of High Pressure Physics, School of Physical Science and Technology, Ningbo University, Ningbo, 315211, People's Republic of China
E-mail: [dumingyang@nbu.edu.cn](mailto:dumingyang@nbu.edu.cn) , [liuzhao@nbu.edu.cn ,](mailto:cuitian@nbu.edu.cn,) [cuitian@nbu.edu.cn ,](mailto:cuitian@nbu.edu.cn,) [weixinmiao@nbu.edu.cn ,](mailto:cuitian@nbu.edu.cn,) (C. Author).

Defang Duan
College of Physics, Jilin University, Changchun 130012, People's Republic of China

Keywords: Hydrides, High pressure, Superconductivity, First principles calculation


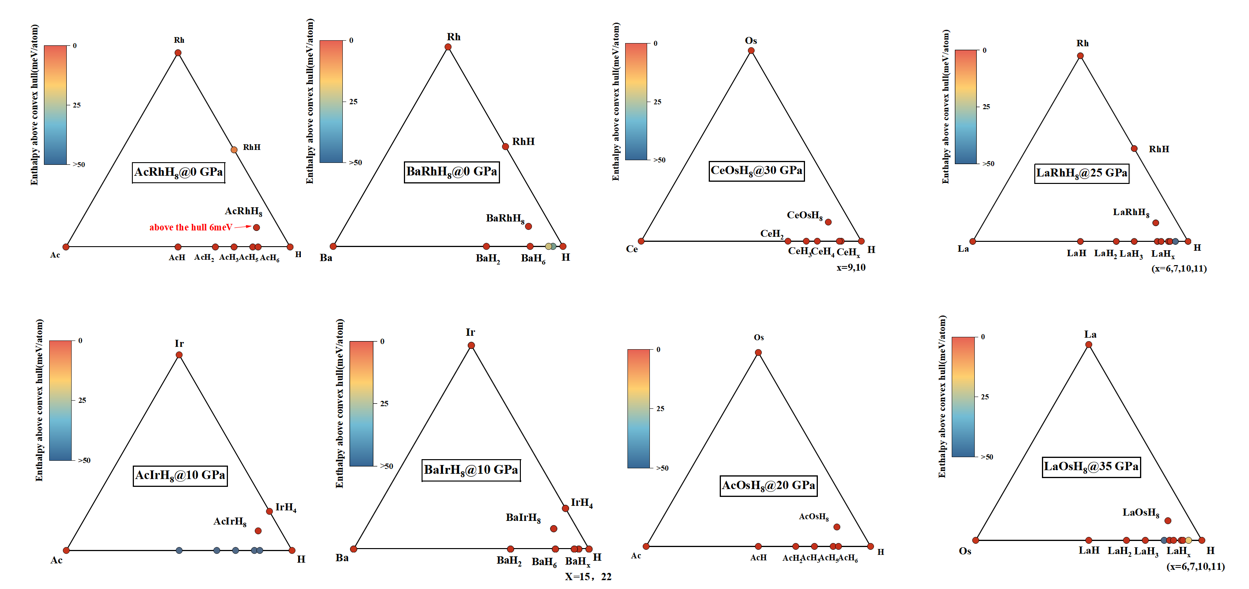
**Figure S1** The ternary phase diagrams (convex hull) of (a) AcRhH_8_ at 0 GPa, (b) AcIrH_8_ at 10 GPa, (c) AcOsH_8_ at 20 GPa, (d) CeOsH_8_ at 30 GPa, (e) BaRhH_8_ at 0 GPa, (f) BaIrH_8_ at 10 GPa, (g) LaRhH_8_ at 25 GPa, and (h) LaOsH_8_ at 35 GPa.

**Table S1** the Bader charges of AXH_8_s at their lowest dynamical stable pressures(unit: e). Negative δ mean loss of electrons, positive δ mean gain of electrons.

| Atomic species | AcRhH_8_ | AcIrH_8_ | AcOsH_8_ | BaRhH_8_ | BaIrH_8_ | LaRhH_8_ | LaOsH_8_ | CeOsH_8_ |
| --- | --- | --- | --- | --- | --- | --- | --- | --- |
| H | 0.23 | 0.24 | 0.17 | 0.18 | 0.16 | 0.19 | 0.22 | 0.23 |
| δ(X)(Rh,Ir,Os) | 0.14 | 0.14 | 1.02 | 0.02 | 0.04 | 0.18 | -0.04 | -0.05 |
| δ(A)(Ac,Ba,La) | -2.02 | -2.04 | -2.40 | -1.50 | -1.36 | -1.75 | -1.71 | -1.76 |

**Table S2** the ELF at the junction and *T_c_*s of Elishaberg of AXH_8_s at their lowest dynamical stable pressures.

| Structures | Pressures | λ | *T_c_*_Elishaberg(K)  μ^*^= 0. 1 | ELF at  The junction |
| --- | --- | --- | --- | --- |
| AcRhH_8_ | 0 | 1.5 | 77.62 | 0.22 |
| AcIrH_8_ | 3 | 1.3 | 63.90 | 0.22 |
| AcOsH_8_ | 17 | 1.1 | 66.35 | 0.2 |
| BaRhH_8_ | 0 | 0.8 | 51.65 | 0.19 |
| BaIrH_8_ | 3 | 1.2 | 67.82 | 0.22 |
| LaRhH_8_ | 24 | 2.2 | 94.28 | 0.3 |
| LaOsH_8_ | 35 | 1.1 | 83.01 | 0.25 |
| CeOsH_8_ | 31 | 1.4 | 106.04 | 0.27 |


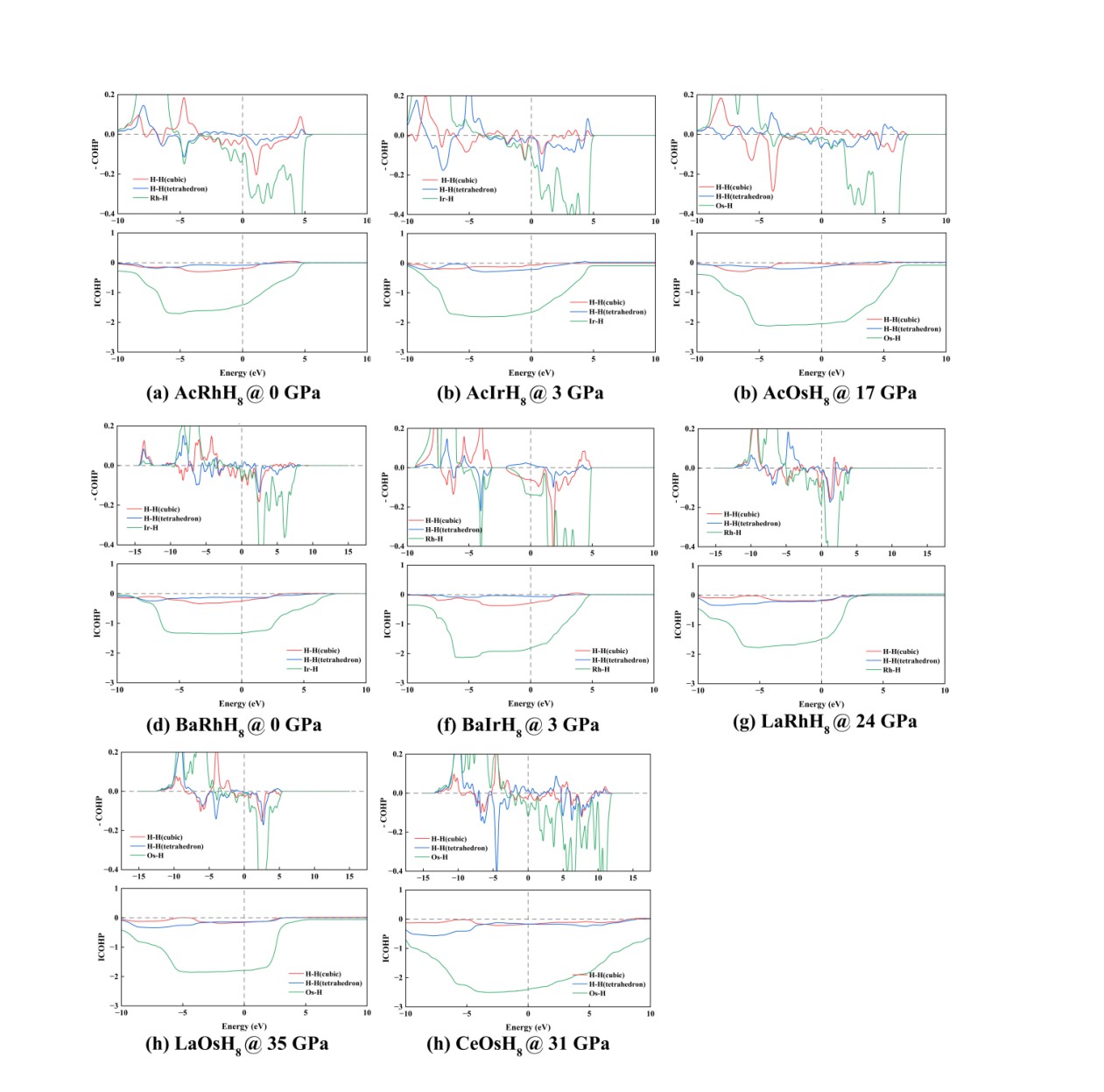


**Figure S2** The COHP of (a) AcRhH_8_ at 0 GPa, (b) AcIrH_8_ at 10 GPa, (c) AcOsH_8_ at 20 GPa, (d) CeOsH_8_ at 30 GPa, (e) BaRhH_8_ at 0 GPa, (f) BaIrH_8_ at 10 GPa, (g) LaRhH_8_ at 25 GPa, and (h) LaOsH_8_ at 35 GPa.


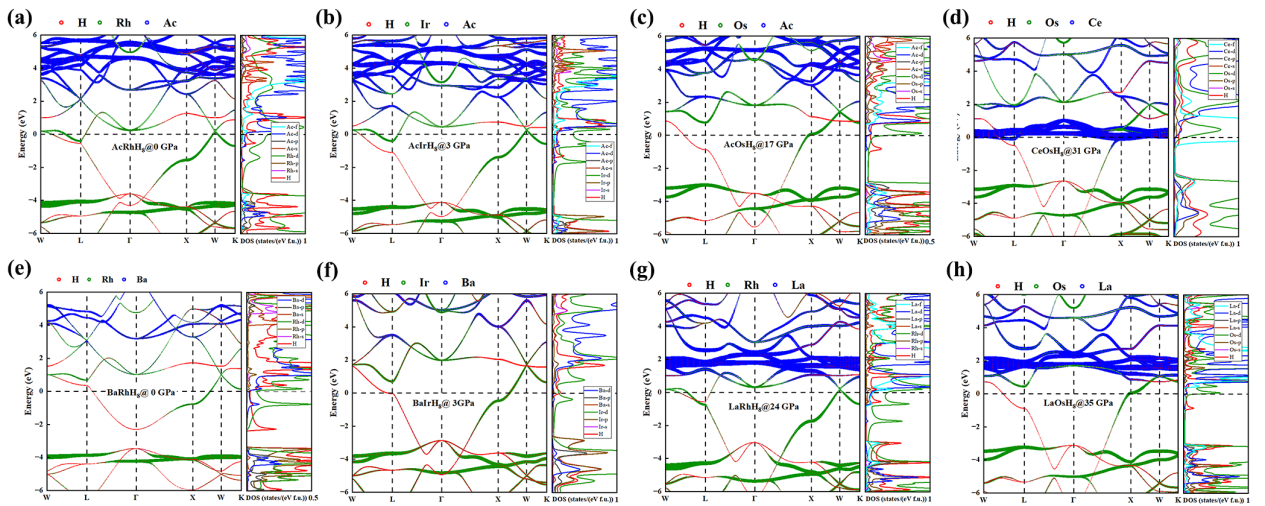
**Figure S3** The electronic fat band structure and orbital projected electronic density of States of (a) AcRhH_8_, (b) AcIrH_8_, (c) AcOsH_8_, (d) CeOsH_8_, (e) BaRhH_8_, (f) BaRhH_8_, (g) LaRhH_8_, and (h) LaOsH_8_ at their lowest dynamical stable pressures.


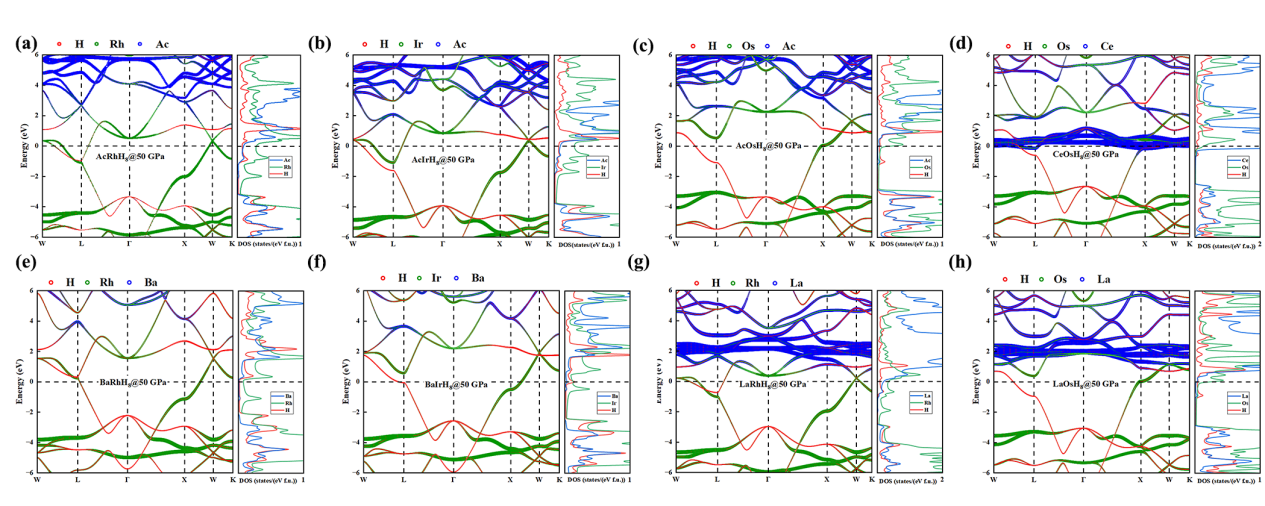


**Figure S4** The electronic fat band structure and the projected electronic density of states of (a) AcRhH_8_, (b) AcIrH_8_, (c) AcOsH_8_, (d) CeOsH_8_, (e) BaRhH_8_, (f) BaRhH_8_, (g) LaRhH_8_, and (h) LaOsH_8_ at 50 GPa.

**Table S3**: The calculated EPC parameter λ, logarithmic average phonon frequency ω_log_ (K) the superconducting critical temperature *T_c_* of AXH_8_s at their lowest dynamical stable pressures.

| Structures | Pressures | λ | ω_log_  （K） | *T_c_*_McM  (K)  μ*=0.1 | *T_c_*_McM  (K)  μ*=0.15 | *T_c_*_AD  (K)  μ*=0.1 | *T_c_*_AD  (K)  μ*=0.15 | *T_c_*_Elishaberg  (K)  μ^*^= 0. 1 | *T_c_*_eliashberg  (K)  μ*=0.15 |
| --- | --- | --- | --- | --- | --- | --- | --- | --- | --- |
| AcRhH_8_ | 0 | 1.5 | 425.6 | 48.42 | 41.31 | 56.59 | 56.00 | 77.62 | 63.90 |
| AcIrH_8_ | 3 | 1.3 | 360.2 | 36.44 | 29.78 | 41.94 | 41.19 | 63.90 | 44.79 |
| AcOsH_8_ | 17 | 1.1 | 694.2 | 56.51 | 44.21 | 61.36 | 60.38 | 66.35 | 50.67 |
| BaRhH_8_ | 0 | 0.8 | 992.4 | 46.78 | 32.24 | 50.40 | 48.44 | 51.65 | 35.48 |
| BaIrH_8_ | 3 | 1.2 | 558.6 | 51.83 | 42.45 | 57.40 | 57.40 | 67.82 | 53.12 |
| LaRhH_8_ | 24 | 2.2 | 357.9 | 54.41 | 54.44 | 67.00 | 69.73 | 94.28 | 81.54 |
| LaOsH_8_ | 35 | 1.1 | 885.0 | 69.51 | 48.77 | 76.82 | 74.32 | 83.01 | 65.37 |
| CeOsH_8_ | 31 | 1.4 | 794.4 | 83.77 | 70.43 | 94.47 | 94.05 | 106.04 | 88.89 |

**
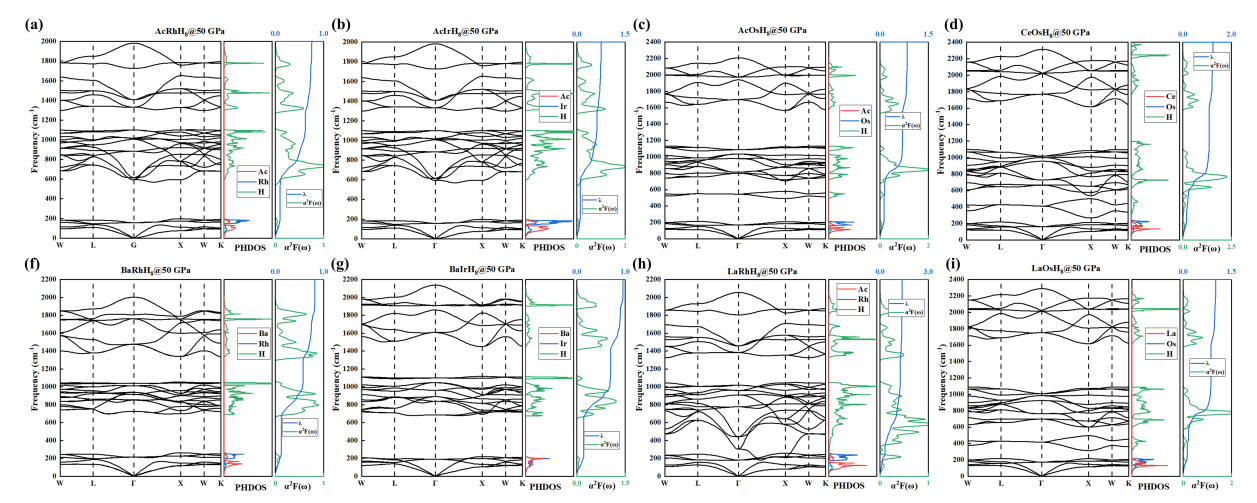
**

**Figure S5** The calculated phonon band structure, PHDOS, EPC parameter λ, and Eliashberg spectral function α^2^F(ω) of (a) AcRhH_8_, (b) AcIrH_8_, (c) AcOsH_8_, (d) CeOsH_8_, (e) BaRhH_8_, (f) BaRhH_8_, (g) LaRhH_8_, and (h) LaOsH_8_ at 50 GPa.
